# *Aspergillus fumigatus* biofilms: Toward understanding how growth as a multicellular network increases antifungal resistance and disease progression

**DOI:** 10.1371/journal.ppat.1009794

**Published:** 2021-08-26

**Authors:** Kaesi A. Morelli, Joshua D. Kerkaert, Robert A. Cramer

**Affiliations:** Department of Microbiology and Immunology, Geisel School of Medicine at Dartmouth, Hanover, New Hampshire, United States of America; Rutgers University, UNITED STATES

## Abstract

*Aspergillus fumigatus* is a saprophytic, filamentous fungus found in soils and compost and the causative agent of several pulmonary diseases in humans, birds, and other mammals. *A*. *fumigatus* and other filamentous fungi grow as networks of filamentous hyphae that have characteristics of a classic microbial biofilm. These characteristics include production of an extracellular matrix (ECM), surface adhesion, multicellularity, and increased antimicrobial drug resistance. *A*. *fumigatus* biofilm growth occurs in vivo at sites of infection, highlighting the importance of defining mechanisms underlying biofilm development and associated emergent properties. We propose that there are 3 distinct phases in the development of *A*. *fumigatus* biofilms: biofilm initiation, immature biofilm, and mature biofilm. These stages are defined both temporally and by unique genetic and structural changes over the course of development. Here, we review known mechanisms within each of these stages that contribute to biofilm structure, ECM production, and increased resistance to contemporary antifungal drugs. We highlight gaps in our understanding of biofilm development and function that when addressed are expected to aid in the development of novel antifungal therapies capable of killing filamentous fungal biofilms.

## Introduction

*Aspergillus fumigatus* is a filamentous, saprophytic fungus that grows as long, branched multinucleate filamentous cells termed “hyphae.” These hyphae associate into a mycelium that is classically defined as a mass of interwoven, and, in some cases, interconnected vegetative hyphae. Mycelia are a common growth form of filamentous fungi and are necessary for the growth of plant mycorrhizal fungi and lichens. The term “mycelia” has long been used to describe the hyphal growth of fungi, and it can be found in case studies published as early as 1898 [1]. Recently, it has been appreciated that the fungal mycelium, in some conditions, has characteristics similar to bacterial biofilms [2–4]. For example, a fungal mycelium is a complex, dense, multicellular network that is embedded in or adhered to a surface, coated in an extracellular matrix (ECM), and exhibits emergent properties characteristic of bacterial biofilms with recalcitrance to antimicrobial drug treatments of particular clinical significance [5]. However, a mycelium is also distinct from sessile bacterial and yeast biofilms in structure. Consequently, while not without some controversy, the use of the term “biofilm” to describe filamentous fungal mycelia has recently become accepted, if not common, in the filamentous fungal pathogenesis community [6–8]. Biofilm is now also used to describe fungal communities in the context of industrial scale filamentous fungal fermentations [3,9]. There seems to be no question that key concepts, such as antimicrobial drug resistance, associated with the term “biofilm” are particularly useful in understanding the form and function of a filamentous fungal mycelia in clinical settings [8,10,11]. However, it is important to recognize the uniqueness of the multicellular filamentous fungal mycelium to fully understand its role in pathogenesis, disease progression, and in response to antimicrobial therapy. Thus, the goal of this review is to discuss our current understanding of the form and function of *A*. *fumigatus* biofilms and identify key gaps in knowledge for further study.

The topic of *A*. *fumigatus* biofilms is important because while *A*. *fumigatus* is most commonly found in soils and compost, it grows as biofilms on mammalian cells in vitro and in vivo [5,12]. Not only has there been an increase in the number of individuals with disease caused by *A*. *fumigatus*, but also treatment options for these diverse diseases remain limited [11]. Moreover, in addition to emerging triazole antifungal drug resistance, infections with antifungal susceptible strains still frequently fail to respond to treatment in vivo [13]. It seems likely that part of the reason for reduced in vivo efficacy of contemporary antifungal therapy is the drug-resistant nature of *A*. *fumigatus* biofilms [5,14]. Current antimicrobial susceptibility testing methods and most drug discovery screening approaches against *A*. *fumigatus* and related molds unfortunately focus on the conidial form of the organism, which is critical for infection initiation but typically not present at the site of infection in the context of established disease that requires antifungal therapy.

Despite a recent increase in the recognition of the clinical importance of fungal biofilms, and years of research on filamentous fungal development and growth in model fungi, such as *Neurospora crassa* and *Aspergillus nidulans*, there are significant gaps in *A*. *fumigatus* biofilm knowledge. The cellular mechanisms orchestrating biofilm formation, structure, and function remain to be precisely described, leaving clinicians at a disadvantage when trying to treat established *A*. *fumigatus* infections where fungal biofilms are present. In order to form biofilms, a population of *A*. *fumigatus* conidia undergoes a series of developmental steps. Throughout biofilm development, emergent properties begin to appear as structure, and distinct microenvironments within the biofilm are established. Consequently, different hyphae, or even sections of a single hyphae, are in distinct physiological states within a filamentous fungal biofilm. Arguably, defining these distinct developmental programs and the resulting intra-biofilm cell heterogeneity is key to better understanding *A*. *fumigatus* biofilm form and function.

**Fig 1:**
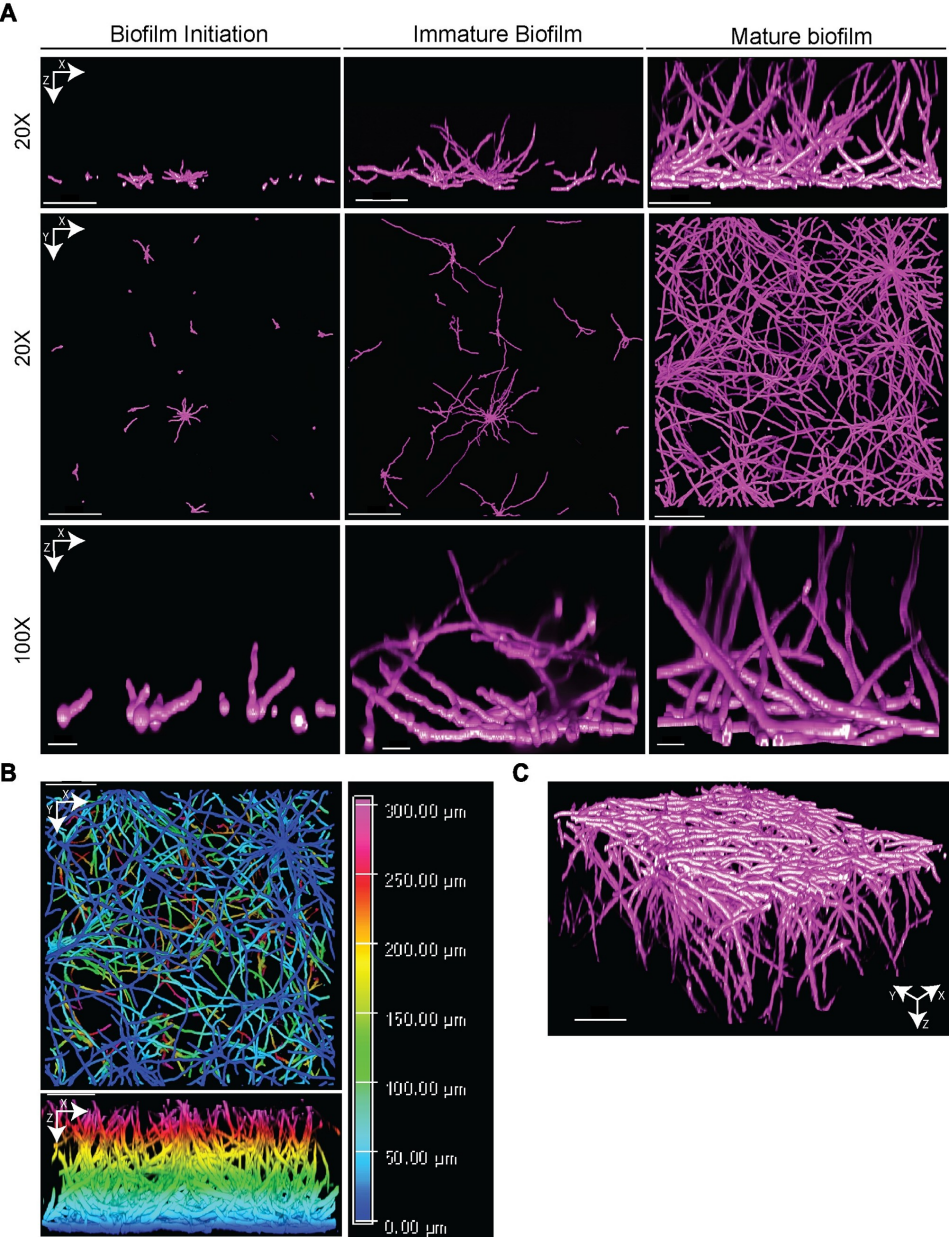
Overview of *Aspergillus fumigatus* biofilm development. **(A)** Representative fluorescence confocal microscopy images of *A*. *fumigatus* CEA10 submerged biofilms grown in liquid minimal media during key stages of development. **(B)** Representative XY and XZ views of 24-hour *A*. *fumigatus* submerged biofilm in minimal liquid media color coded to show height of hyphae within the biofilm. **(C)** XYZ view of 24-hour *A*. *fumigatus* submerged biofilm in liquid minimal media. The 20× magnification scale bar is 100 um. The 100× magnification scale bar is 10 um.

There are currently 2 commonly used models for studying *A*. *fumigatus* biofilm development in vitro that have distinct characteristics [11]. *A*. *fumigatus* biofilms occur in vitro on agar surfaces as colony biofilms or in liquid cultures as submerged biofilms [2,4]. A key distinction between these 2 biofilms is the propensity for agar surface cultures to rapidly initiate asexual reproduction and abundant production of asexual conidia. It is worth considering that much of our understanding of filamentous fungal development and stress responses has come from the study of agar surface-based cultures. However, one significant advantage of the submerged biofilm model is the ability to interrogate stress responses at different stages of biofilm development and maturity. Moreover, the submerged model allows quantitative imaging of biofilm development and architecture (e.g., **Fig 1**). These advantages, plus the observation that asexual development is rarely observed in vivo during invasive aspergillosis, led us to focus this review on 3 distinct stages of *A*. *fumigatus* submerged biofilm formation. These 3 stages are initiation, immature biofilm, and mature biofilm. We review features at each stage of biofilm development as well as key genes and their role in biofilm development (**Table 1**). The future of highly efficacious antifungal treatments will be built on a better understanding of filamentous fungal biofilm biology.

**Table 1:** Summary of *Aspergillus fumigatus* genes discussed in this review.

Gene name	AF293 Gene ID	Function	Reference(s)
*gprK*	Afu4g01350	Mediates germination in response to carbon sources. Null mutants have increased germination in the absence of carbon	[26]
*sfaD gpgA*	Afu5g12210 Afu1g05210	GPCR subunits, null mutants have delayed germination	[23]
*sakA*	Afu1g12940	*A*. *fumigatus* HOG1 homolog. Induced in response to nitrogen starvation, null mutants have increased germination with poor nitrogen sources, null mutants have decreased adhesion	[29,71]
*srbA*	Afu2g01260	Hypoxia responsive bHLH transcription factor essential for growth in low oxygen. Regulates sterol synthesis genes, has a role in polarity, and is required for biofilm structure and maturation	[31,86,88,14]
*pkac1 pkac2*	Afu2g12200 Afu5g08570	Protein kinases that regulate oxidative metabolism. Null mutants have reduced germination	[32]
*rasA*	Afu5g11230	Regulates stages of conidial germination in response to carbon sources	[40]
*crzA*	Afu1g06900	Mediator of calcineurin signaling null mutants have reduced germination	[41]
*calA*	Afu3g09690	Binds host integrin to facilitate invasion of host tissue	[55,56]
*uge3*	Afu3g07910	UDP-glucose epimerase required for GAG synthesis. Null mutants are unable to adhere to abiotic surfaces and exhibit reduced adherence to epithelial cells	[66]
*medA*	Afu2g13260	Transcription factor that regulates GAG biosynthetic cluster. Null mutants have reduced *uge3* expression and reduced adhesion	[67]
*stuA*	Afu2g07900	Transcription factor that regulates GAG biosynthetic cluster. Null mutants have reduced *uge3* expression and reduced adhesion	[67]
*somA*	Afu7g02260	Transcription factor, upstream regulator of *medA* and *stuA*. Role in GAG biosynthesis regulation. Null mutants have decreased adherence	[68]
*mpkA*	Afu4g13720	MAP kinase, null mutants have decreased adherence, cell wall integrity	[71]
*sitA*	Afu6g11470	MpkA-regulating phosphatase, upstream regulator of *mpkA* in coordinating surface adhesion and cell wall composition	[72]
*mdr4*	Afu1g12690	Multidrug resistance pump up-regulated in maturing biofilms	[79]
*cdr1B*	Afu1g14330	Putative drug efflux pump	[81]
*atrR*	Afu2g02690	Transcription factor, regulates *cdr1B* expression. Null mutants have significantly reduced expression of *cdr1B*. Low oxygen growth defect	[81]
*cyp51A*	Afu4g06890	14-α sterol demethylase, null mutants have increased azole sensitivity	[85]
*bafA*	Afu5g14915	Cryptic gene sufficient to induce hypoxia morphology	[95]

### Biofilm initiation: Conidial swelling, germination, and adhesion

The first stage of *A*. *fumigatus* submerged biofilm formation takes place over the first 12 hours of culture after inoculation of conidia, but exact timing depends on the specific culture conditions. The most commonly used submerged biofilm culture model is grown in 24- or 96-well polystyrene plates under standard laboratory conditions [2,14,15]. We have broadly termed the first 12 hours of biofilm development “biofilm initiation.” Unlike the initiation of many model bacterial biofilms where transition from motile to a nonmotile state is a crucial defining step, the initiation of *A*. *fumigatus* biofilm formation is largely dependent on the conidia adhering to a surface and undergoing a series of developmental events that leads to the emergence of hyphae [16,17]. Previous research has defined adhesion, swelling, and germination as separate and distinct stages of biofilm initiation; however, swelling and germination occur at a single-cell level rather than a community level, and adhesion is not necessarily restricted to a specific morphological stage of the fungus. Thus, while these are distinct biological processes (discussed below), we suggest that together they constitute biofilm initiation. Throughout this stage of initiation, the population largely lacks a high-order structure, has minimal secreted ECM, and cells are still susceptible to external stresses such as antifungal drug treatment [15].

#### Conidial swelling and germination

Swelling, or isotropic growth of conidia, is the first step of biofilm initiation [18]. In *Aspergillus* spp., conidial swelling is triggered by suitable conditions that include, but are not limited to, nutrient availability, favorable temperatures, and sufficient oxygen [19–21]. Importantly, it has been shown that conidial density has a significant impact on the formation of a stable, drug resistant in vitro submerged biofilm [2]. While the exact mechanisms that trigger swelling in response to these environmental conditions are unknown in *A*. *fumigatus*, some of the sensors have been identified in *A*. *nidulans*. In *A*. *nidulans*, temperature is sensed by TcsB and FphA, where FphA modulates the expression of many downstream genes via the HOG signaling pathway [22]. RasA and cAMP mediated signaling initiate conidial swelling in response to carbon sources in *A*. *nidulans* [20,23]. However, RasA overactivation does not alter production of cAMP, suggesting that these signaling pathways act independently of each other [24]. Upstream of cAMP signaling, in *A*. *nidulans* surface G protein–coupled receptors (GPCRs), such as GprH, regulate initiation of germination in response to glucose [25].

In *A*. *fumigatus*, strains lacking the GPCR *gprK* had 80% germinated conidia compared to wild-type conidia, which had 35% germination at 8 hours of growth in the presence of carbon [26]. Without a carbon source, Δ*gprK* mutants had approximately 50% germination at 16 hours compared to wild-type conidia, which had less than 10% germination, indicating that GprK plays a role in regulating germination in response to carbon sensing. The GPCR subunits SfaD and GpgA also play a role in GPCR-mediated germination [27]. *sfaD* and *gpgA* null strain conidia begin germination between 12 and 14 hours of incubation, whereas wild-type and complemented strains germinate between 4 and 6 hours. In addition to glucose, inorganic phosphate, inorganic nitrogen, or magnesium sulfate are also required for initiation of germination in static *Aspergillus niger* cultures [28]. SakA, the *A*. *fumigatus* HOG1 homolog, is transcriptionally activated in response to nitrogen starvation, while the *ΔsakA* mutant strain has increased germination when grown in minimal media containing poor nitrogen sources (such as sodium nitrate) when compared to more preferred nitrogen sources (such as proline) [29].

**Fig 2:**
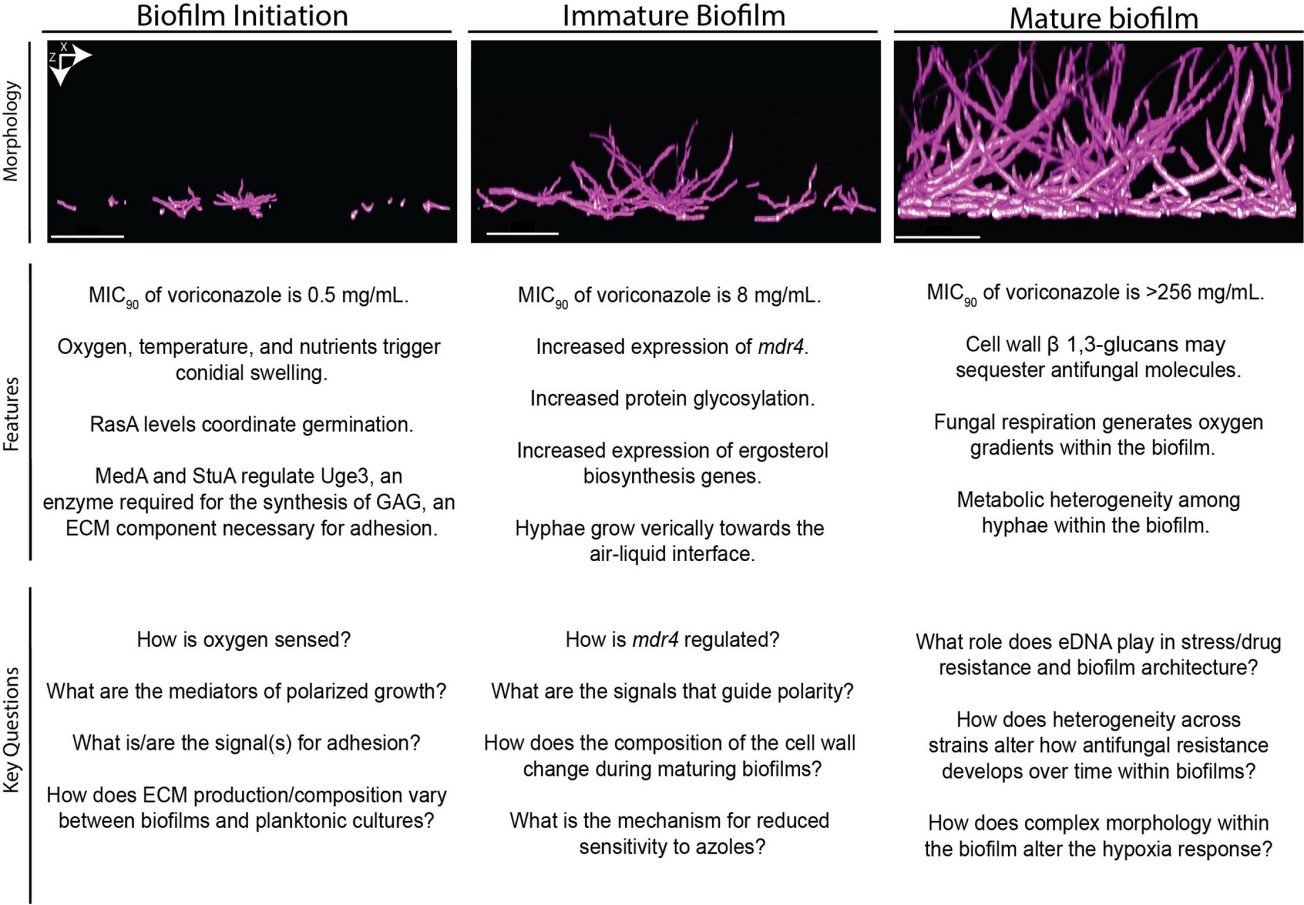
Summary of key stages of biofilm development. Summary of each stage of biofilm development showing XZ view of a representative fluorescence confocal microscopy image of *A*. *fumigatus* CEA10 submerged biofilms. Here, we summarize features of each stage as well as key questions that remain to be answered. All images are 20× magnification. Scale bar is 100 um. ECM, extracellular matrix; eDNA, extracellular DNA; MIC, minimum inhibitory concentration.

Oxygen is also required for conidial activation and germination [21]. When incubated in anoxia, conidia stay dormant, and do not germinate until they are moved to 21% oxygen [30]. Additionally, a strain lacking *srbA*, a transcription factor essential for growth in low oxygen conditions, has hyphal growth in environments with 21% oxygen but not at 1% oxygen environments [31]. This result suggests that *A*. *fumigatus* conidia sense differences in oxygen tension to mediate germination. Currently, there are no known mechanisms of direct oxygen sensing in any *Aspergillus* species. It is possible that the activation of germination in response to oxygen is an indirect mechanism mediated by oxidative phosphorylation. Although this idea has not been directly tested, deletion of the kinases PkaC1 and PkaC2, which regulate the expression of genes involved in oxidative metabolism, results in reduced germination [32]. Thus, how oxygen impacts conidia activation remains an important area of *A*. *fumigatus* biofilm initiation research (**Fig 2**).

Similarly, physical contact may also play a role in conidial germination via thigmotropism (response to touch). While there are no proteins annotated to contribute to thigmotropism in the *Aspergillus* genome, mechanisms utilized by other pathogenic fungi, such as the filamentous plant fungal pathogens, may be insightful to understand how *A*. *fumigatus* conidia coordinate germination and polarized growth in response to specific surfaces. For example, in plant pathogenic fungi such as *Magnaporthe* spp., the ends of hyphae form into an invasive structure termed the appressorium [33]. In *Magnaporthe grisea*, this process is coordinated via Pth11p-mediated GPCR signaling [34]. In the *pth11* null mutant, germinated conidia have 10% to 15% appressorium formation when compared to wild type. It would be interesting to examine *pth11* mutants biofilm initiation phenotypes in fungi such as *A*. *fumigatus* that do not generate appressoria.

Once activated, conidia break dormancy, and isotropic swelling initiates. Transcriptional analysis of swollen conidia in *A*. *fumigatus* revealed that mRNA from approximately half of all genes within the gene ontology (GO) category “RNA binding and translational regulation” were induced in conidia incubated in nutrient rich media for 30 minutes [35]. In contrast, only about 15% of genes involved in transcriptional regulation were observed in swollen conidia. Genes involved in amino acid metabolism, ribosome biogenesis, protein metabolism, and posttranslational modification are thus significantly enriched in swollen conidia. The coordination of increased translational activation and cell growth has been observed in both *Saccharomyces cerevisiae* and *Candida albicans* and is regulated via the HOG pathway [36,37]. These findings agree with previous observations that conidia contain prepackaged mRNA to expedite protein synthesis upon the initiation of swelling [38]. Finally, in swollen conidia, one-third of all genes in the stress response and oxidoreductase GO categories were down-regulated [35]. This highlights how conidia transition from a quiescent, stress resistant state in favor for a more metabolically and translationally active state necessary for active growth.

Germination of conidia begins around 4 to 8 hours, depending on the culture conditions and strain, and is the next step of *A*. *fumigatus* biofilm initiation. Germination is a 4-step process that begins with the aforementioned breaking of dormancy and conidial swelling followed by the initiation of polarized hyphal growth and emergence of a germ tube (**Fig 1A**) [39]. Active RasA is required for conidial swelling in response to favorable conditions but is inhibitory to germ tube development [40]. High levels of active RasA results in swollen conidia that fail to germinate until they are switched to conditions that favor intermediate RasA activity. The zinc finger transcription factor CrzA, a downstream mediator of calcineurin signaling, is also important at this stage of germination [41]. Strains lacking *crzA* had only 6% germinated conidia at 8 hours compared to a *crzA* complemented strain, which had 48% germinated conidia. The *ΔcrzA* strain had a defect in polarized growth and decreased glucan synthase in hyphae, suggesting that CrzA regulates germination and polarized growth through the biosynthesis of cell wall components. There are likely other signals that specifically mediate the polarized growth stage of germination, but conidial germination assays are often based only on the quantification of the percent of conidia, which have formed a germ tube—the last step in germination. This method lacks temporal precision and does not differentiate between signaling pathways necessary for polarized growth versus those required for the exit of dormancy. Therefore, there are still significant gaps in our knowledge of the pathways specifically required for the establishment of polarized growth and the formation of a germ tube. Cutting-edge flow cytometric analyses of conidia activation may be a high-throughput tool to identify key genes involved in the exit from dormancy.

As mentioned, conidia inoculum density has a significant impact on the formation of an *A*. *fumigatus* biofilm. The inoculum density requirement raises the potential for quorum sensing in *A*. *fumigatus* biofilm initiation. For bacterial biofilms, quorum sensing is a method of cell-to-cell communication that plays a major role in adherence, biofilm formation, colonization, and virulence [42–44]. In fungi, including species of *Aspergillus*, inoculum size has been observed to alter growth rate, morphology, and inhibitory concentrations of certain antifungals [39,45,46]. Thus, it is plausible that there are cell-to-cell communications utilized by *A*. *fumigatus* to mediate biofilm formation in response to cell density. While these mechanisms remain to be identified in *A*. *fumigatus*, quorum sensing mechanisms that mediate cell growth have been observed in other fungi. For example, *C*. *albicans* cultures grown in static conditions produce extracellular farnesol to mediate hyphal growth [47]. In *Cryptococcus neoformans*, an 11-amino acid quorum sensing–like protein, QSP1, facilitates growth in low density cultures in a *tup1* null strain [48]. Given the importance of cell-to-cell communication in bacterial biofilm assembly and evidence for quorum sensing in other fungi, it would be beneficial to determine whether quorum sensing is utilized by *A*. *fumigatus* and how intercellular communication might play a role in biofilm formation. Here, one wonders about a potential role for the abundant secondary metabolites produced by *A*. *fumigatus* and other filamentous fungi [49].

#### Conidial adhesion

Once conidia exit dormancy, they are capable of adhering to many surfaces ranging from plastic culture dishes to human epithelial and endothelial cells [50,51]. Adhesion of swollen and germinating conidia is likely reliant on surface adhesins as well as the production and secretion of a polysaccharide rich ECM. ECM-mediated adhesion of hyphae will be discussed in detail below for immature biofilms.

Initial adhesion of inert conidia to culture dishes is thought to be caused by weak, temporary electrostatic interactions [39]. However, once dormancy is broken, the hydrophobic outer layer of the conidia cell wall is broken down, exposing surface adhesins and cell wall α-glucans [52]. Approximately 80 genes in the *A*. *fumigatus* genome encode adhesin or adhesin-like proteins and about 30 of these genes have been classified as conidia specific [53]. Despite several putative adhesins being identified both bioinformatically and biochemically, the role of surface adhesins in biofilm formation, adherence, and general *A*. *fumigatus* physiology remains largely under explored. Biochemical studies have revealed that conidia can bind to host laminin and fibronectin in a protein mediated manner, but the responsible proteins remain to be determined [54]. The protein CalA was found to bind laminin, but its observed protein size does not match that of the original laminin binding study, suggesting that multiple proteins have the potential to bind laminin [55]. CalA also binds to integrin on host cells to induce endocytosis, therefore facilitating invasion of host tissue [56]. The role of CalA in invasive growth emphasizes the importance of identifying and characterizing genes encoding adhesin and adhesin-like proteins.

Hydrophobins on conidia are one group of proteins known to be involved in surface interactions. Hydrophobic rodlet proteins in the cell wall of conidia mediate binding to collagen, another host ECM protein abundant in the lung [57]. Additionally, biochemical studies have characterized a fucose binding lectin and a sialic acid binding lectin present on the surface of *A*. *fumigatus* that both have hemagglutination activity [58–60]. Alpha-glucans on the other hand have been shown to be relatively dispensable for vegetative growth on agar plates but are essential for adherence of swelling conidia to each other [61,62]. While adherence of conidia to other conidia is not a prerequisite for biofilm formation, it has potential implications for biofilms forming in environments where flow forces or stochastic disruptive forces, such as those caused by coughing, are present. Thus, while some biochemical and genetic groundwork has been laid, there is a need to further explore the players and mechanisms of fungal adherence in different contexts of *A*. *fumigatus* biofilm formation.

### Immature biofilms: ECM, antifungal resistance, and structure

At 12 hours of incubation in glucose-rich culture conditions, germlings have elongated into hyphae that crisscross over one another forming a monolayer at the bottom surface. These immature biofilms are a transitional phase in biofilm growth defined by moderate ECM production, cell wall changes, signs of increasing structure (such as hyphal branching), and increased antifungal drug resistance compared to the populations of conidia discussed above. Thus, despite this being an active phase of biomass generation, by 12 hours, immature biofilms have decreased sensitivity to all 3 classes of antifungal drugs when compared to 8-hour cultures [15]. Next, we discuss key players and features of these immature biofilms.

#### ECM-mediated adhesion

The seminal discovery of *A*. *fumigatus* ECM production was a major step toward applying the biofilm concept to filamentous fungi [4,5,12]. ECM extracted from static grown *A*. *fumigatus* biofilms analyzed by nuclear magnetic resonance spectroscopy (NMR) was found to mostly consist of glycans and proteins but also contain small amounts of lipids, aromatics, and extracellular DNA (eDNA) [63]. While these experiments, conducted in Roswell Park Memorial Institute (RPMI) medium, give an example of ECM composition, they do not fully explore the complexity of the *A*. *fumigatus* ECM and how it changes under different environmental conditions. In *C*. *albicans* biofilms, matrix production is in part facilitated by endosomal sorting complexes required for transport (ESCRT)-mediated extracellular vesicles (EVs), and the individual deletion of 7 different ESCRT genes resulted in decreased ECM production [64]. Since *A*. *fumigatus* mycelia produce EVs future experiments assessing *A*. *fumigatus*, EV production within biofilms and their contribution to matrix production may be worthwhile [65].

In *A*. *fumigatus* mutants lacking *uge3*, a UDP-glucose epimerase required for galactosaminogalactan (GAG) synthesis, a major component of the ECM, germlings were unable to adhere to culture dishes and exhibited less than 10% adherence to epithelial cells when compared to wild-type germlings [66]. In an immunosuppressed mouse model of invasive aspergillosis, the *Δuge3* mutant was found to have attenuated virulence with increased murine survival at 8 days post-fungal challenge compared to wild type [66]. GAG also functions to conceal surface β[1,3]-glucan, which may contribute to the decreased virulence observed for strains lacking *uge3*. While the exact kinetics of ECM secretion are not fully explored under different environmental and substrate conditions, ECM secretion likely begins shortly after swelling in order to facilitate surface adhesion. The signals and underlying mechanisms of ECM regulation at these early stages of filamentous fungal biofilm development remain to be fully defined.

Expression of the GAG biosynthetic gene cluster containing *uge3* is regulated by the 3 transcription factors, MedA, StuA, and SomA [66–68]. Loss of MedA or StuA transcriptional regulators results in reduced expression of *uge3*, little to no detectable GAG, and reduced adhesion [66,67]. SomA regulates GAG synthesis through the regulation of MedA and StuA and by direct binding to the promoter regions of the GAG biosynthesis genes *agd3* and *ega3* [68,69]. In the m*edA* null mutant, the expression of 8 putative adhesins was compared to wild type and one, Afu3g00880, was found to be regulated by MedA [67]. To date, this gene has yet to be validated as an adhesin and further highlights a key gap in knowledge regarding protein-based adhesins important for biofilm formation and structure. The interplay between protein-based adhesins and the ECM-mediated adherence remains to be fully defined. It is unclear how ECM composition may change under different conditions and under what conditions and in what contexts protein-based adhesins may play an important role in surface adherence (**Fig 2**). A complete catalog of transcription factors required for adhesion to diverse surfaces under a variety of conditions is now possible with the release of an *A*. *fumigatus* whole genome transcription factor deletion collection [70]. Broadening our understanding of the key transcriptional regulatory circuits involved in adhesion is expected to yield new insights into fungal biofilm formation.

To this end, SakA and MpkA are 2 MAP kinases involved in *A*. *fumigatus* surface adhesion. *sakA* and *mpkA* null mutants had less adhesion when grown in liquid minimal media in fibronectin treated plates as quantified by a crystal violet (CV) assay [71]. Germlings of an MpkA null mutant have 50% less GAG, while SakA null germlings have 50% more GAG when compared to WT strains when quantified using the Soy Bean Agglutinin (SBA) lectin staining [71]. SBA preferentially binds oligosaccharides with terminal alpha or beta-linked *N-*acetylgalactosamine residues and galactose residues to a lesser extent. The MpkA-regulating phosphatase SitA also plays a key role in regulating cell wall composition and surface adhesion [72]. Upstream mediators of SitA as well as transcription factors downstream of SakA and MpkA that mediate this adherence phenotype are currently unknown. However, the SakA regulated transcription factor AtfA is critical for conidia stress resistance and germination [73]. In *C*. *albicans*, Mkc1p, the MpkA homolog, is phosphorylated in a contact-dependent manner, suggesting that this mechanism may also occur in *A*. *fumigatus* but has yet to be fully explored [74]. The CV assays with Δ*mpkA* and Δ*sakA* were performed using mature biofilms so future experiments assaying the adhesion of Δ*mpkA* and Δ*sakA* germlings are needed to better understand the role of these kinases in adhesion during germination and immature biofilm development. Conceptually, investigating the signals responsible for the activation of these MAP kinases in the context of surface adhesion will give us greater insight into mechanisms of *A*. *fumigatus* biofilm formation.

#### Antifungal resistance of immature biofilms

From 8 to 12 hours, the voriconazole minimum inhibitory concentration (MIC_90_; the concentration of voriconazole needed to reduce cell viability by 90%) increases from 0.5 mg/L to 8 mg/L [15,75]. How does this remarkable increase in MIC for antifungal drugs arise at this stage of fungal biofilm development? These data also raise an important consideration for in vivo drug treatments; even an immature filamentous fungal biofilm displays antifungal drug resistance through an unknown mechanism(s). A potential explanation for decreased drug susceptibility throughout biofilm formation might be the increased expression of multidrug resistance (MDR) efflux pumps. MDR efflux pumps are ATP-dependent transporters, which belong to the larger ATP-binding cassette (ABC) transporter family. ABC transporters as well as major facilitator superfamily (MFS) transporters, the second major class of transporters found in *A*. *fumigatus*, actively transport a variety of molecules, such as the drug voriconazole, to remove them from the cell [76]. The *A*. *fumigatus* genome contains 49 predicted ABC transporters as well as 278 predicted MFS proteins, of which 35 are putative multidrug permeases [77,78]. Transcripts for the ABC-type transporter encoding gene *mdr4* are significantly increased at 12 hours compared to 8 hours when grown in vitro in RPMI media [79]. This increase in *mdr4* transcription in 12-hour biofilms compared to 8-hour biofilms also correlated with a significant increase in drug resistance. Additionally, random mutagenesis of *A*. *fumigatus* conidia identified mutants that were highly resistant to the antifungal itraconazole, which had increased basal expression *mdr3* and *mdr4* or increased expression in the presence of itraconazole in shaking cultures when compared to wild type [80]. Future experiments assessing the azole susceptibility of an *mdr4* mutant strain are crucial to better understanding the relationship between *mdr4* expression and antifungal resistance within biofilms. Induction of transporters may indicate a complex interplay between individual hyphal cell metabolism and microenvironmental niches that remain to be explored. As efflux pumps are important for removing a variety of compounds, it is possible that these pumps are up-regulated in order to remove toxic molecules that might become concentrated in biofilms and serendipitously aid in antifungal resistance as a result. For example, biofilms grown on an agar surface down-regulate glycolysis in favor of alternate metabolic pathways that may lead to the buildup of potentially toxic metabolic byproducts [79]. Metabolite profiling of the extracellular biofilm environment at different stages of development may yield new insights into fungal biofilm metabolism and subsequent stress resistance phenotypes.

Further studies defining regulators of drug efflux pumps in biofilms will provide much needed insight into biofilm mediated antifungal resistance at this early stage of biofilm development (**Fig 2**). Transcriptional or posttranslational MDR pump regulation remains under-explored in *A*. *fumigatus*. The putative drug efflux pump Cdr1B has been shown to be regulated by the transcription factor AtrR [81]. In a Δ*atrR* strain, expression of *cdr1B* was reduced to less than 10% of wild-type expression. However, the role of AtrR and Cdr1B in *A*. *fumigatus* biofilms remains to be defined. Direct examination and genetic analyses of additional specific transporters are needed, but these studies may be confounded by the substantial redundancy of pump function. An overexpression-based approach may reveal new key efflux pump-based mediators of biofilm antifungal drug sensitivity, but the power of transcription factor and protein kinase mutants to identify candidate effector genes should not be overlooked.

Physical barriers, such as the aforementioned ECM or cell wall, are a likely contributor to fungal biofilm antifungal drug resistance and tolerance, as observed with cell wall glucan-dependent drug resistance of *C*. *albicans* biofilms [82]. Cell wall changes are dynamic, robust, and occur rapidly as conidia swell, germinate, and transition to hyphae. Cell wall differences between strains has been linked to virulence, showing how the cell wall plays an important role in infection and disease outcome [83]. Transcriptional analysis of colony biofilm (on agar surface) cultures suggest that 16-hour biofilms have significantly more protein glycosylation than hyphae in planktonic (free floating in liquid) cultures [7]. This increased glycosylation results in “sticky” proteins, which may glue hyphae together within the biofilm and could conceivably make biofilms less permeable to drugs.

Regarding the ECM, studies have intriguingly revealed that *A*. *fumigatus* Δ*uge3* biofilms, lacking the ECM component GAG, do not have decreased antifungal resistance at 18 hours when compared to wild-type biofilms [14]. However, antifungal treatment of 9-hour *A*. *fumigatus* biofilms with the addition of hydrolases capable of degrading GAG showed significantly decreased MIC_50_ for all 3 classes of antifungals [84]. This highlights the importance of GAG during biofilm initiation while also illustrating how other factors besides GAG contribute to antifungal resistance at later stages of biofilm development. How other ECM components contribute to antifungal resistance will be covered later in this review when we discuss antifungal resistance within mature biofilms.

A comparison of shaking planktonic and static colony biofilm cultures found increased expression of ergosterol biosynthesis genes in colony biofilms. Deletion of the gene *cyp51A*, which encodes for a 14-α sterol demethylase, has been shown to increase sensitivity to azoles [85]. This observation raises an intriguing question regarding the mechanism for enhanced ergosterol gene expression in biofilms and whether this transcriptional increase corresponds to alterations in sterol levels within the fungal cells at this point in biofilm development. Sterol biosynthesis is directly impacted by iron and oxygen levels and reductions in these key molecules stimulates ergosterol synthesis gene expression through SrbA-dependent mechanisms [86]. In submerged biofilm cultures, oxygen levels are depleted over the course of biofilm development and correspond with an increase in transcription of the SrbA dependent gene *erg25A* [14]. Thus, as biofilm cell density increases, oxygen levels within the immature biofilm become depleted leading to both an increase in expression of ergosterol biosynthetic genes and a decrease in metabolism. These 2 factors may contribute to the localized increases in antifungal resistance within the developing biofilm. This hypothesis remains to be tested.

#### Polarized growth and morphology

Lastly, a mechanism important for the morphology of immature biofilms is cell polarity, which serves to guide hyphae as they grow to form an interconnected mycelium. Filamentous fungi grow in a polarized manner, directing growth at the tip via formation of a vesicle rich structure called the Spitzenkörper [87]. Consequently, regulators of fungal cell polarity machinery such as endocytosis and cytoskeletal elements are likely important for biofilm form and function but remain largely unstudied in the context of submerged biofilms. Seminal discoveries of genes involved in these processes in model organisms such as *N*. *crassa* and *A*. *nidulans* can serve as a foundation for studies in *A*. *fumigatus*. Here, the question of developmental programs comes into play. Are these vertical growing hyphae under static liquid submerged culture conditions a result of asexual development initiation? Or do they remain in a vegetative state consuming and probing for new nutrient sources such as oxygen (**Fig 2**)?

In static liquid cultures with adequate nutrients and oxygen, *A*. *fumigatus* hyphae consistently form a basal mycelium followed by vertical hyphal growth (**Fig 1A**) [3,14]. This vertical polar growth toward the surface is seen in immature biofilms by 12 hours [14]. Intriguingly, SrbA, which is required for growth in low oxygen conditions in *A*. *fumigatus*, has been shown to be involved in cell polarity and microtubule dynamics in *Aspergillus* spp. [31,88]. Consequently, it is interesting to note that oxygen availability clearly alters biofilm architecture. In static liquid cultures, the morphology of biofilms changes with oxygen availability [14]. Hyphae within *A*. *fumigatus* submerged biofilms grown in 21% oxygen are mostly vertical, while hyphae grown under a 5% oxygen atmosphere or less intriguingly grow more laterally or horizontally. *A*. *fumigatus* strains that are more fit in low oxygen conditions also exhibit altered biofilm morphology at higher oxygen concentrations with an increase in the percentage of filaments that grow horizontally [83]. These data suggest connections between cell polarity, biofilm morphology, and oxygen availability and pose important questions. For example, while oxygen availability alters biofilm morphology, the signaling pathways that mediate this process are unknown. Furthermore, the connection between oxygen availability and cell polarity remains to be defined. These questions highlight the importance of understanding and better characterizing the immature, transitional stage of biofilm development.

### Mature biofilms: High antifungal resistance, increased complexity, and heterogeneity

**Fig 3:**
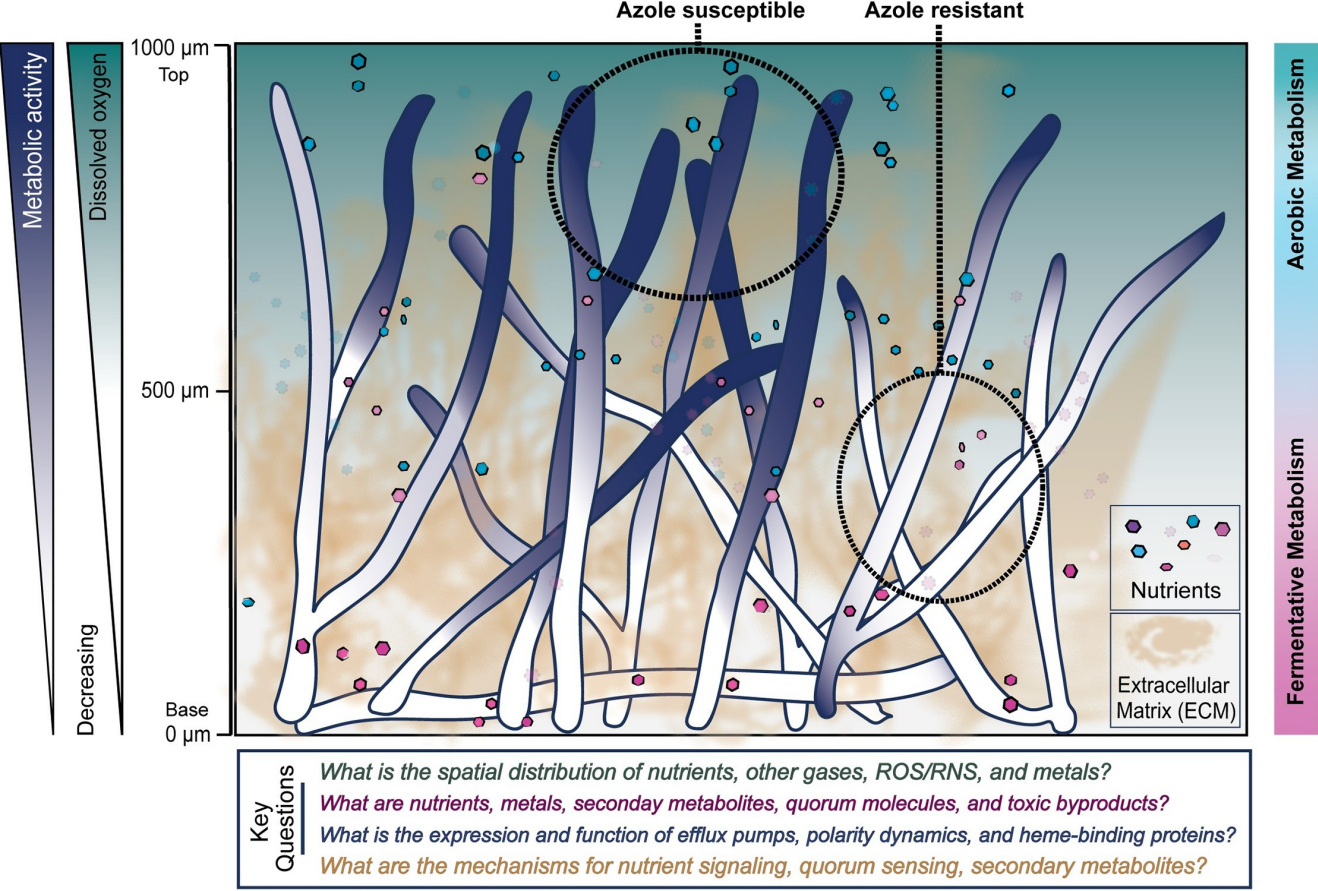
Model of mature *A*. *fumigatus* biofilms. Illustration depicting known contributors to *A*. *fumigatus* mature biofilm complexity. Gradients of oxygen and metabolic activity develop along a vertical axis within the biofilm which likely creates a shift from respiratory metabolism to fermentative or other alternative metabolisms at the bottom of the biofilm due to oxygen depletion. This change in metabolic activity also plays a key role in azole resistance at the base of the biofilm. ECM coats hyphae and collects at the base of the biofilm. Key questions remain about the spatial distribution of certain molecules, the identities of things such as quorum sensing molecules, the expression or function within biofilms of cellular components such as mediators of polarity, and the signaling mechanisms for processes like quorum sensing. ECM, extracellular matrix; RNS, reactive nitrogen species; ROS, reactive oxygen species.

At 24 hours post-inoculation, antifungal drug efficacy is strikingly decreased in mature submerged biofilms compared with immature biofilms, with the MIC_90_ of voriconazole increasing from 8 mg/L at 12 hours to an astounding >256 mg/mL at 24 hours [15]. Therefore, we will refer to submerged biofilms of 24 hours or older as mature biofilms. These biofilms are best characterized as having complex structure, exhibiting high levels of antifungal resistance, vast quantities of ECM, and steep oxygen and nutrient gradients (**Fig 3**). The morphological, transcriptional, and metabolic changes that occur during biofilm maturation remain to be fully defined. Defining a standardized time point of analysis and key markers for biofilm maturation should be a goal of the field moving forward.

#### Complexity and antifungal resistance

The buildup of ECM material peaks at 36 hours in static cultures of *A*. *niger* grown in potato dextrose broth on polystyrene dishes, encasing the fungus in a protective layer that glues hyphae together [89]. As mentioned previously, in *C*. *albicans*, ECM serves as a physical barrier to antifungals through glucan binding and sequestration of antifungals [82]. It has also been shown that the ECM of *C*. *albicans* also acts as a physical barrier to host leukocytes and is capable of inhibiting extracellular trap formation by neutrophils (NETs) [90]. In *A*. *nidulans*, increased production of GalNAc-rich GAG through the overexpression of *ugeB* or heterologous expression of *A*. *fumigatus uge3*, increased resistance to neutrophil NET formation in vitro [91]. How mature *A*. *fumigatus* biofilms impact immune effector cell function remains to be fully defined and is an important area of investigation moving forward.

In addition to polysaccharides, the presence of eDNA in the ECM has been reported at 24 to 48 hours in *A*. *fumigatus* biofilms [92]. eDNA not only supports fungal biofilm architecture but also aids in antifungal resistance [92]. In static cultures, the concentration of amphotericin B and caspofungin needed to inhibit fungal growth decreased as the biofilms were treated with increasing concentrations of DNAse, suggesting a functional role for eDNA release in biofilms. Previous studies in both bacteria and *Aspergillus* spp. suggests that eDNA is released via autolysis, a process that likely occurs within growing biofilms as they adapt to their environment [93,94]. Still, the exact mechanism of eDNA secretion into the ECM is unknown, and the extent to which this occurs under conditions relevant in the infection environment is unclear (**Fig 2**).

#### Macroscopic colony morphology and virulence

Exposure to low oxygen also results in changes in macroscopic colony biofilm morphology, such as increased colony furrows and a white perimeter of vegetative growth, on solid media [83]. Some strains have a hypoxia-locked morphology, termed “H-MORPH,” where colony biofilm morphology resembles low oxygen culture conditions even in oxygen-replete conditions. H-MORPH has been associated with increased mortality in a murine model of invasive aspergillosis [83]. A single cryptic gene, *bafA*, is sufficient to induce H-MORPH in both the *A*. *fumigatus* reference strains AF293 and CEA10 and the *A*. *niger* reference strain A1144 [95]. Overexpression of *bafA* in these strains alters both macroscopic and microscopic biofilm morphology and decreases adhesion of submerged biofilms. Interestingly, *bafA* has several putative orthologs, including a pseudogene, which display copy number variation across strains of *A*. *fumigatus*. Moreover, *bafA* was discovered in the sub-telomeric region of chromosome 5 of strain AF293, a region of novel and rapidly evolving genes [96]. Sub-telomeric region genes’ mRNA levels were significantly induced in the agar colony biofilm model [79]. However, the molecular function of *bafA* remains unknown and it is unclear how its expression impacts biofilm morphology. Yet, the example of *bafA* also highlights that many uncharacterized fungal specific genes found in sub-telomeric regions have important and uncharacterized roles in fungal biology.

#### Heterogeneity within biofilms

Evidence of metabolic heterogeneity within a mature biofilm has been observed, but it is not yet understood how and to what extent these metabolic changes contribute to biofilm form and function. For example, fungal respiration reduces available oxygen within the biofilm, resulting in zones of oxygen depletion by 18 hours of growth in the submerged biofilm model (**Fig 3**) [14]. Hyphae at the base and toward the interior of the biofilm are thus likely in an altered metabolic state driven in part by reduced oxygen availability compared to peripheral cells. The interplay between localized oxygen depletion and alterations in metabolism throughout the biofilm remains to be defined experimentally. Importantly, hyphae in regions of oxygen limitation are strikingly resistant to antifungal drugs. Azoles, in particular, such as voriconazole, require metabolic activity to effectively target ergosterol synthesis (**Fig 3**). Curiously, planktonic grown hyphae or conidia are not more resistant to antifungals in an atmosphere with reduced oxygen availability [14]. This raises intriguing questions about the hypoxia response in planktonic versus submerged biofilm conditions in fungi that remain to be defined. Presumably, carbon and nitrogen sources available to the fungus in the interior of the biofilm are also altered compared to exterior and planktonic cells, further impacting the metabolism of these oxygen limited regions. Yet, studies addressing these questions are currently in their infancy with filamentous fungal biofilms. In addition, the low oxygen environment inside the biofilm might inhibit the generation of reactive oxygen species (ROS) by neutrophils and confer resistance to leukocyte mediated killing but remains to be experimentally tested for filamentous fungal biofilms.

As *A*. *fumigatus* biofilms mature and oxygen gradients are established, the complexity of these biofilms increases. As we better understand biofilm growth, the need for spatial resolution becomes more apparent. For example, the decreased expression of efflux pumps at 24 hours may not be a global change within biofilms, but rather a result of altered metabolic activity in different regions of the biofilm. Oxygen gradients are just one example of important macro and micronutrient gradients that may become established over time in biofilms. Additional factors such as other gases, nutrients, secondary metabolites, host-generated molecules, and host cell infiltration may also occur regionally within biofilms to impact their function and maturation (**Fig 3**). The use of reporter strains is one tool that can be used to investigate spatial heterogeneity in these filamentous fungal biofilms. The development and application of other tools to further spatially dissect fungal biofilms and focus on one region at a time will greatly increase our understanding of biofilm form and function.

### Strain heterogeneity

Finally, an underutilized resource for defining and understanding the form and function of filamentous fungal biofilms is the natural heterogeneity of *A*. *fumigatus* strains. Not only does overall biofilm morphology vary, as seen in H-MORPH strains, but also altered biofilm morphology correlates with increased virulence [83]. This raises questions concerning how differences in both macroscopic and microscopic biofilm morphology confer advantages to different strains in vivo and if these differences are related to antifungal resistance or leukocyte inhibition. While decreased susceptibility to antifungal drugs as biofilms develop has consistently been reported for frequently used lab strains, this is not true for all strains of *A*. *fumigatus*. When submerged biofilms from a selection of lab strains and environmental isolates of *A*. *fumigatus* were treated with voriconazole at 12 and 18 hours, one environmental isolate was found to be significantly more susceptible at 18 hours [14]. Further examination of the heterogeneity across strains could give important insights into the morphological and physiological characteristics which play a role in antifungal drug resistance. Moreover, large-scale genomics and transcriptomics studies should be used to identify genes that respond to biofilm conditions to identify key regulators and mediators at different stages of biofilm development [97].

### Methods for studying *A*. *fumigatus* biofilm development

In general, methods to assess submerged biofilm formation and function can be divided into 2 broad categories, population level assays for growth and/or viability, and direct visualization of biofilms using microscopy. Since *A*. *fumigatus* grows as a biofilm in static liquid cultures, laser nephelometry and optical density (OD) growth curves can be used to assess broad differences in biofilm initiation. While OD is commonly used due to its wide availability, laser nephelometry has proven itself to be more appropriate for the type of structured filamentous growth present in filamentous fungal biofilms [98]. Thus, while less readily available, laser nephelometry can be used as a high-throughput and more accurate measurement of submerged filamentous fungal biomass during biofilm development. Dry biomass is the most direct method for quantifying growth, but it is a relatively low throughput method and in some media the polysaccharide rich ECM can become a confounding variable. A less direct, but high-throughput, method of specifically assessing adherent biomass is the CV assay. The high number of manipulations involved in the CV assay, combined with the strong adherence of *A*. *fumigatus* to abiotic surfaces, allows for non-adherent and weakly adherent biomass to be removed and only the adherent biomass to be assayed.

Metabolic dyes, such as Resazurin and XTT, are used as an indirect method for assessing growth and viability. When comparing between different strains, however, any basal metabolic differences can be a confounding variable in the use of metabolic dyes. Thus, metabolic dyes are best used to assess the impact of a drug or stress treatment on a given biofilm. The method of applying stress to a forming or mature biofilm can be compared to the conidial or pre-biofilm susceptibility of a strain to differentiate the emergent properties specific to the biofilm and the genetically encoded resistance of a strain to a particular stress. However, new methods to monitor the viability and fitness of a given submerged biofilm in response to specific stresses are needed. Here, the use of specific viability and stress reporter strains may prove fruitful [14].

The ability to examine fungal biofilms at a high resolution using confocal and scanning electron microscopy (SEM) is particularly powerful for the structural and functional characterization of biofilms [39,83]. Combining these high-resolution techniques with ever advancing image processing software, including the open-source Fiji and BiofilmQ softwares, allows for sophisticated quantification of biofilms at both the population and micron scale [99,100]. SEM has proven itself indispensable in understanding high resolution biofilm structure of many organisms, including some of the first characterizations of *A*. *fumigatus* biofilms [5,39]. Aspects of the ECM can also be directly visualized via SEM; however, the process of preparing samples of SEM requires dehydration of the sample and thus yields specific artifacts. While also having some caveats, confocal microscopy is highly versatile and can be utilized to acquire spatially resolved information on both living biofilms and endpoint assays. Utilizing constitutively fluorescent strains one can directly visualize living biofilms supporting experimentation over the time of a biofilm’s development. The use of fluorescent dyes that stain the entire hyphae, such as calcofluor white, can also be utilized to image biomass of non-fluorescent strains, although usually as an endpoint metric as many of these dyes can also cause cell wall stress. Use of fluorescent biomass markers in combination with fluorescent dyes, antibodies, and promoter fusions, where expression of a fluorescent protein is driven by the promoter of a gene of interest, can provide detailed spatial information on physiological aspects of the biofilm.

One major need for the field with regards to microscopy is the integration of microfluidics devices where the environment can be highly controlled and spatially restricted, given that in vivo biofilms are, at least initially, restricted to the space provided by the airways. While microfluidics devices have been successfully used to visualize individual hyphae, their use with *A*. *fumigatus* biofilms have yet to be successfully performed. This is, in part, due to technical challenges associated with cheaply making devices of the size scale necessary to accommodate *A*. *fumigatus* biofilms and a need to better understand the initial adherence of *A*. *fumigatus* to abiotic surfaces. Finally, recent reports of utilizing spinning disk confocal microscopy and light-sheet microscopy in combination with tissue clearing techniques allowed the direct imaging of fungal biofilms within infected murine lungs in 3 dimensions [83,101,102]. These techniques are technically challenging, but continued improvement on them and the implementation of various dyes and antibodies would provide exceptional levels of insight into phenomena that are occurring in vivo during disease progression.

### Biofilms among bacterial communities

Lastly, an understudied mode of *A*. *fumigatus* biofilm growth is the development of biofilms in overlapping niches with bacterial communities. Not only does *A*. *fumigatus* grow among commensal bacteria within the lungs of immunocompromised individuals, but also *A*. *fumigatus* is common in persons with cystic fibrosis who also have bacterial infections [103,104]. A study of 201 cystic fibrosis patients found that nearly 60% were colonized with *A*. *fumigatus*, and, of those patients colonized with *A*. *fumigatus*, about 80% were also colonized with *Pseudomonas aeruginosa* [105]. A more recent study found that *Aspergillus spp*. were culturable from about 32% of sputum samples from nearly 2,600 cystic fibrosis patients [106].

Studies of *A*. *fumigatus* grown with bacteria are in the early stages, but preliminary work has shown the complexities of these *A*. *fumigatus* bacterial communities. *P*. *aeruginosa* has been found to secrete short chain carbon molecules, such as decanoic acid, which inhibit *A*. *fumigatus* biofilm formation [107]. In contrast, it has also been suggested that *P*. *aeruginosa* colonization within the lung may favor *A*. *fumigatus* sensitization, allowing for fungal growth within the lung [108]. Both *A*. *fumigatus* and *P*. aeruginosa produce siderophores to compete for limited nutrients such as iron [109,110]. Experiments incubating *A*. *fumigatus* with supernatant from *P*. *aeruginosa* found that pyoverdine, a siderophore produced by *P*. *aeruginosa*, is an inhibitor of *A*. *fumigatus* growth [111]. This study of iron scavenging, performed with supernatant rather than in coculture, begins to characterize the complex interactions between these microorganisms in vitro. Cocultures of *P*. *aeruginosa* and *A*. *fumigatus* established for the extraction of metabolites give insights into the conditions, such as media and inoculum, necessary for successful cocultures [112]. Overall, much remains to be learned about the complexity of bacterial and fungal cocultures in disease-relevant settings.

## Conclusions

Much clearly remains to be learned about each stage of *Aspergillus* biofilm development. Improving our understanding of *A*. *fumigatus* biofilm form and function will require melding insights from basic fungal biology with clinically relevant models of antifungal drug and immune cell exposures. Examining early signals and genes that mediate adhesion and germination will give insight into the fundamental steps of biofilm formation. These early stages are potential therapeutic targets for prophylactic treatments to perhaps prevent disease initiation in susceptible patient populations. Transcriptional changes in cell wall components and efflux pumps give insight into important mechanisms necessary for this stage of development. However, most *A*. *fumigatus* infections are not diagnosed until more mature biofilms have already been established within the lung. At the final stages of biofilm growth, *A*. *fumigatus* becomes an intricate network of filaments with a dense secreted ECM and regions of limited oxygen. This makes both successful immune cell responses and antifungal treatment difficult and calls for more studies defining the important spatial metabolic differences within biofilms. Finally, cross-species interactions between bacteria and *A*. *fumigatus* biofilms are generally lacking despite their relevance to diseases such as cystic fibrosis. However, the groundwork for studying *A*. *fumigatus* biofilms has been laid over the past 2 decades. In the end, a more complete understanding of *A*. *fumigatus* biofilm biology is expected to uncover therapeutic opportunities to enhance contemporary antifungal drug susceptibility and/or perhaps identify new antifungal molecules with biofilm specific mechanisms of action.
